# 2040 Symptom endorsement in bipolar patients of African Versus European ancestry

**DOI:** 10.1017/cts.2018.263

**Published:** 2018-11-21

**Authors:** Margaret Akinhanmi, Joyce E. Balls-Berry, Suliman El-Amin, Jennifer Geske, Colin Colby, Christi Patten, Joanna Biernacka, Mark A. Frye

**Affiliations:** Mayo Clinic

## Abstract

OBJECTIVES/SPECIFIC AIMS: Learning Objectives of this session: Identify possible reasons for misdiagnosis of bipolar patients of African ancestry by reviewing differences in symptom presentation between African American (AA) and European American (EA) bipolar individuals. Introduction: Bipolar disorder is a chronic mental illness with a prevalence rate up to 5.5% of the US population and is associated with substantial personal and economic morbidity/mortality. Misdiagnosis is common in bipolar disorder, which can impact treatment and outcome. Misdiagnosis disproportionally affects racial/ethnic minorities; in particular, AAs are often misdiagnosed with schizophrenia. There is interest in better understanding the contribution of differential illness presentation and/or racial bias to misdiagnosis. METHODS/STUDY POPULATION: Patients and Methods Utilizing the Genetic Association Information Network (GAIN) public database, this study compared clinical phenomenology between bipolar patients of African Versus European ancestry (AA=415 vs. EA=1001). The semi-structured Diagnostic Interview for Genetic Studies (DIGS) was utilized to evaluate individual symptom endorsement contributing to diagnostic confirmation. A χ^2^ test was used to compare group differences in DIGS harvested mania and psychosis sections, and overview of psychiatric medications. RESULTS/ANTICIPATED RESULTS: Results: The symptom of auditory hallucination was significantly more endorsed in AA bipolar patients than EA bipolar patients (57.9% AA vs. 36.1% EA, *p*≤0.0001). Conversely, the symptom of elevated or euphoric mood was significantly less endorsed in AA bipolar patients than in EA patients (94.6% AA vs. 97.5% EA, *p*=0.027). AA, in comparison to EA bipolar patients, had a significantly higher prevalence of lifetime exposure to haloperidol (36.9% AA vs. 29.4% EA, *p*=0.017) and fluphenazine (12.3% AA vs. 6.7% EA, *p*=0.004). In contrast, AA, in comparison to EA bipolar patients, had a significantly lower prevalence rate of lifetime exposure to lithium (52.5% AA vs. 74.2% EA, *p*<0.0001), and lamotrigine (13.7% AA vs. 35.6% EA, *p*<0.0001). DISCUSSION/SIGNIFICANCE OF IMPACT: Conclusion: The higher rate of psychotic symptom endorsement and lower rate of core manic symptom endorsement represent differential illness presentation that may contribute to misdiagnosis in African-American bipolar patients. The higher rate of high potency typical antipsychotic treatment and lower rate of classic mood stabilizing treatment may also contribute poorer bipolar treatment outcome. While structured diagnostic interviews are the gold standard in diagnostic confirmation, this study is limited by lack of knowledge of clinician/expert interviewer interpretation of symptom endorsement which may contribute to symptom misattribution and misdiagnosis. Incorporation of additional African American participants in research is a critical future direction to further delineate symptom presentation and diagnosis to serve as validation for these results.

